# Effect of K-N-Humates on Dry Matter Production and Nutrient Use Efficiency of Maize in Sarawak, Malaysia

**DOI:** 10.1100/tsw.2010.121

**Published:** 2010-07-06

**Authors:** Auldry Chaddy Petrus, Osumanu Haruna Ahmed, Ab Majid Nik Muhamad, Hassan Mohammad Nasir, Make Jiwan

**Affiliations:** ^1^ Department of Crop Science, Faculty of Agriculture and Food Sciences, Universiti Putra Malaysia Bintulu Sarawak Campus, Bintulu, Sarawak, Malaysia; ^2^ Department of Forest Management, Faculty of Forestry, Universiti Putra Malaysia, Serdang, Selangor, Malaysia; ^3^ Department of Animal Science and Fishery, Faculty of Agriculture and Food Sciences, Universiti Putra Malaysia Bintulu Sarawak Campus, Bintulu, Sarawak, Malaysia

**Keywords:** sago waste, humic substances, humin, maize, nitrogen, potassium

## Abstract

Agricultural waste, such as sago waste (SW), is one of the sources of pollution to streams and rivers in Sarawak, particularly those situated near sago processing plants. In addition, unbalanced and excessive use of chemical fertilizers can cause soil and water pollution. Humic substances can be used as organic fertilizers, which reduce pollution. The objectives of this study were to produce K- and ammonium-based organic fertilizer from composted SW and to determine the efficiency of the organic-based fertilizer produced. Humic substances were isolated using standard procedures. Liquid fertilizers were formulated except for T2 (NPK fertilizer), which was in solid form. There were six treatments with three replications. Organic fertilizers were applied to soil in pots on the 10th day after sowing (DAS), but on the 28th DAS, only plants of T2 were fertilized. The plant samples were harvested on the 57th DAS during the tassel stage. The dry matter of plant parts (leaves, stems, and roots) were determined and analyzed for N, P, and K using standard procedures. Soil of every treatment was also analyzed for exchangeable K, Ca, Mg, and Na, organic matter, organic carbon, available P, pH, total N, P, nitrate and ammonium contents using standard procedures. Treatments with humin (T5 and T6) showed remarkable results on dry matter production; N, P, and K contents; their uptake; as well as their use efficiency by maize. The inclusion of humin might have loosened the soil and increased the soil porosity, hence the better growth of the plants. Humin plus inorganic fertilizer provided additional nutrients for the plants. The addition of inorganic fertilizer into compost is a combination of quick and slow release sources, which supplies N throughout the crop growth period. Common fertilization by surface application of T2 without any additives (acidic and high CEC materials) causes N and K to be easily lost. High Ca in the soil may have reacted with phosphate from fertilizer to form Ca phosphate, an insoluble compound of phosphate that is generally not available to plants, especially roots. Mixing soil with humin produced from composted SW before application of fertilizers (T5 and T6) significantly increased maize dry matter production and nutrient use efficiency. Additionally, this practice does not only improve N, P, and K use efficiency, but it also helps to reduce the use of N-, P-, and K-based fertilizers by 50%.

## INTRODUCTION

The main product of the sago palm (*Metroxylon sagu*) is starch. The state of Sarawak, Malaysia at present is the principal producer of sago, exporting about 25,000 to 40,000 tonnes of sago starch annually[1]. Sago waste (SW), which is copious fibrous residue, is disposed after starch extraction from the sago trunk. For every 100 kg of sago starch in pith (70–90 kg can be extracted), there is 10 kg of waste[2]. In addition, SW is likely to be discarded into rivers because most of the factories are built near riversides. This practice may cause water pollution. In addition, inefficient starch extraction will contribute to large amounts of waste[3]. This quantity may be greater if the extraction efficiency is low as sugars, proteins[2], and starches can create high BOD and COD levels in the river[4]. Some researchers have used SW as a substrate for cultivation of edible mushrooms[5], animal feed, production of enzymes[6] and absorbents[7], to reduce this waste.

From an environmental standpoint, SW can be used for agricultural purposes where it can be composted to obtain organic fertilizer. In addition, both of these outputs may reduce the environmental pollution caused by agricultural waste (e.g., SW) and chemical fertilizers. Composting can be considered as the humification technology[8] that occurs when a large part of original organic matter is mineralized and residual organic matter is transformed into new organic materials called humic substances[9]. Humic substances are known as one of the greatest carbon reservoirs on earth and they are used for agricultural, industrial, environmental, and biomedical activities[10]. Humic substances can be divided into three fractions; namely, humic acids (HAs) (soluble in an alkaline solution), fulvic acids (FAs) (fractionally soluble in an aqueous solution at any pH), and humin (fractionally insoluble)[11]. Humic substances are known to be advantageous for soils to maintain aggregate stability and act as a potential source of nutrients for plants[12,13]. In addition, humic substances have been used in the formulation of organic-based fertilizer to reduce ammonia volatilization[14]. The reaction of humic substances with N-containing compounds, such as urea, which is now widely used as a fertilizer, has considerable economic implications[15] to its slow release source.

Composting and its main attribute, i.e., humic substances, was the focus of this study. The objectives of the study were to: (1) produce organic K- and ammonium-based organic fertilizer from composted SW, and (2) determine the efficiency of the organic-based fertilizer produced.

## MATERIALS AND METHODS

The SW was collected from Mukah, Sarawak, Malaysia. The SW was air dried and some of it was ground for the purpose of ashing, while ungrounded SW was used to produce compost.

Ground SW was incinerated at 300, 350, 400, 450, 500, 550, and 600ºC using a muffle furnace for 5 h in a beaker of 500 mL. The best ash (almost white) was chosen for hydroxide production. The ash was dissolved in distilled water at ratios of 1:100, 1:200, 1:300, 1:400, and 1:500. The samples were equilibrated for 24 h at 150 rpm using a mechanical shaker. Afterwards, the samples were filtered using Whatman filter paper number 2. The molarity was determined using acid-base titration. A ratio of 1:500 was chosen because its hydroxide had the highest molarity of 0.002 M and pH of 10. The chosen hydroxide was analyzed for its mineral content where it was found that Ca (42.88 mg $kg^{-1}$) and K (29.51 mg $kg^{-1}$) were high in the hydroxide compared to other elements.

The composting process was done inside a white polystyrene box with a size of 61.5 × 49 × 33.5 cm. The compost was produced by mixing SW (80%) + chicken feed (10%) + chicken dung slurry (5%) + molasses (5%). The ambient and compost temperatures were measured daily (morning and evening). The temperature of the compost was monitored until it equaled ambient temperature for 60 days, where it was considered mature. Turning was done once a week.

Humic acids were isolated by the method of Stevenson[16], but with some modifications. The compost and the hydroxide (0.002 M) (extracted from the ash of SW) were placed inside a polyethylene bottle in a ratio of 1:10 (weight:volume basis). The mixture was shaken at 240 rpm for 24 h at room temperature. Afterwards, the mixture was centrifuged for 15 min at 10,000 rpm. The dark-colored supernatant liquid containing HA was decanted, filtered using Whatman filter paper number 2, the pH of the liquid was adjusted to 1.0 using 6N HCl, and it was allowed to stand at room temperature for 24 h. The suspension containing HA was transferred into a polyethylene bottle and centrifuged at 10,000 rpm for 10 min. The HA was purified by the method of Ahmed et al.[17], by using distilled water and through centrifugation at 10,000 rpm for 10 min to reduce mineral matter and HCl during acidification[18]. After the purification, the HA was oven dried at 40ºC until constant weight was attained.

The soil of Bekenu series (*Tipik Tualemkuts*) was used for the pot experiment. Samples were taken at a depth of 0–30 cm. Prior to planting, soil characteristics, such as texture, cation exchange capacity (CEC), exchangeable cations (K, Ca, Mg, Na), organic matter, and organic carbon, were determined using the combustion method[19]; pH in water and KCl (1:2.5 w:v); available P using the blue method[20], total N using the Kjeldahl method[21], nitrate and ammonium content[22] were determined.

Production of the organic fertilizer was made through reconstitution of HA with hydroxide obtained from ashed SW (0.002 M). The ratio thereof was set at 1:20, 1:40, 1:60, 1:80, and 1:100 (w:v). These mixtures were then shaken for 24 h at 150 rpm, after which they were analyzed for K, Ca, Mg, and Na using AAS (atomic absorption spectrometry). Afterwards, the 1:40 mixture was found to yield the highest content of minerals (K, Ca, Mg, Na); thus, it was selected for fertilizer production. This liquid HA was designated as T4.

The compost and the hydroxide (0.002 M) (extracted from the ash of SW) were placed inside a polyethylene bottle at a ratio of 1:10 (w:v). The mixture was shaken at 240 rpm for 24 h at room temperature. Later, it was centrifuged for 15 min at 10,000 rpm. The resulting dark liquid containing HAs and FAs was decanted. The mixtures were then used as treatments three (T3) and six (T6).

The humin (in solid form) deposits in the bottles were collected and analyzed for pH, CEC, exchangeable cation (K, Ca, Mg, Na) (using leaching method), total N (using Kjeldahl method), and total minerals (K, Ca, Mg, Na, Cu, Zn, Fe, Mn, P). All minerals were analyzed from the single dry ashing method[23]. All minerals except P were determined using AAS, while P was determined using color development (blue method) and analyzed using UV-vis spectrometer.

The pot experiment was conducted in a greenhouse at the Universiti Putra Malaysia Bintulu Sarawak Campus, using randomized complete block design (RCBD) with three replications. The pots used were in a size of 21.5 × 28 cm. The medium for each treatment consisted of 10 kg soil (based on bulk density of the soil), except for T4 and T5 where the soil had to be reduced to compensate for additional 200 g of humin for T5 and 100 g of humin for T6. The Masmadu variety of maize (*Zea mays*) was used as the test crop. Its fertilizer requirement was 60 kg N, 60 kg $P_2O_5$, and 40 kg K2O (130.44 kg $ha^{-1}$ urea; 130.44 kg $ha^{-1}$ TSP; 66.67 kg $ha^{-1}$ KCl). The fertilizer requirement was scaled down to per-pot basis equivalent to 4.85 g of urea, 4.85 g of TSP (triple superphosphate), and 2.5 g of KCl. The volume of water for each pot was based on field capacity (50–60%). The six treatments devised for this experiment were as follows:

- T1: No fertilization
- T2: NPK (4.85 g urea, 4.85 g TSP, 2.5 g KCl) (solid)
- T3: 400 mL liquid of FA + HA mixed with 4.85 g of urea and 2.5 g of KCl + 4.85 g TSP
- T4: Liquid HA mixed with 4.85 g of urea and 2.5 g of KCl + 4.85 g TSP
- T5: 400 mL Ca-K hydroxide (extracted from ash) mixed with 4.85 g of urea and 2.5 g of KCl + 4.85 g TSP + 200 g humin in soil

T6: 400 mL liquid of FA + HA mixed with 4.85 g of urea and 2.5 g of KCl + 4.85 g TSP + 100 g humin in soil

Treatments T3 to T6 were in liquid form where urea and KCl were added, shaken for 15 min at 150 rpm, and pH measured. TSP for T3, T4, T5, and T6 was applied separately by surface application. It is important to note that the 400-mL solution was chosen as Piccolo[24] recommended, that less than 1 g of HA per kilogram soil is adequate to condition soils. In this study, 10 g of HA was adulterated with 400 mL of hydroxide based on chosen ratio of 1:40, before being applied to 10 kg of soil. Hence, the volume of liquid fertilizer of all treatments was based on the volume of T4. All treatments were applied on the 10th day after sowing (DAS), but on the 28th DAS, only plants of T2 were fertilized. The plants were monitored and their heights were measured until the tassel stage. Tassel stage is the maximum growth stage for the plant before it goes to productive stage[14].

Harvesting was done on the 57th DAS. The whole plant, including leaves and stems, was harvested. The remaining roots in the soil were removed carefully and cleaned using tap and distilled water. The plant parts (leaves, stems, and roots) were then oven dried at 60ºC until constant weight and their dry matter were determined. Soil samples were also taken from the pots and air dried, ground, and sieved to pass 2 mm before being analyzed for pH, total carbon, total nitrogen, organic matter, P, ammonium and nitrate, exchangeable cation (K, Ca, Mg, Na), and CEC using standard procedures. Each plant part was ground and analyzed for total K, Mg, Ca, Na, N, and P.

Nitrogen, P, and K use efficiency were determined by the formula[25]:

$$\text{\% Efficiency} = \frac{A - B}{C} \times 100$$

where A = uptake with fertilizer, B = uptake without fertilizer, C = total amount of fertilizer that had been applied; uptake = concentration × dry weight (g).

Nitrogen, P, and K uptake in leaves, stems, and roots were determined by multiplying the concentration with the dry weight of the plant parts. Analysis of variance (ANOVA) was used to test the effect of treatments, while means of treatments were compared using Tukey’s Test. Statistical Analysis System (SAS Version 9.2) was used for the statistical analysis.

## RESULTS AND DISCUSSION

The selected chemical and physical properties of Bekenu series (*Tipik Tualemkuts*) were relatively different from those reported by Paramanathan[26] (Table 1) because this soil has been cropped for some time.

The relatively high values of the selected chemical properties of the unpurified humin suggest the high quality of this humin fraction for plant growth and development (Table 2). For instance, neutral pH, high organic matter, and CEC of the humin may have loosened the soil as well as improving nutrient retention when applied to soils. Besides serving as a source of nutrients, such as N, P, K, Ca, Mg, and Na, along with good C/N ratio of the humin, it also ensures that immobilization will not occur when it is used in agriculture.

The pH of T3, T4, T5, and T6 did not change significantly before and after addition of urea and KCl (Table 3), suggesting that they were buffered.

The height of plants was monitored from 10 DAS until 57 DAS. Ten days after the first fertilization, the height of plants for T5 and T6 started to increase rapidly compared to other treatments. This was because the humin of T5 and T6 contributed to a slow release of nutrients in T5 and T6. Although both T3 and T6 had liquid HA and FA, the plants of T6 grew better than those of T3 due to the addition of humin to the soil of T6. The inclusion of humin might have loosened the soil, hence the better growth of the plants. Obviously, the plants of T1 were stunted, due to no fertilization.

**TABLE 1**
**Selected Physicochemical Characteristics of Bekenu Series**

| Variables | Value Obtained | Standard Data Range[26] |
|---|---|---|
| pHw | 6.45 | 4.6–4.9 |
| pHKCl | 5.87 | 3.8–4.0 |
| Exchangeable K (cmol $kg^{-1}$) | 0.13 | 0.05–0.19 |
| Exchangeable Na (cmol $kg^{-1}$) | 0.06 | 0.01 |
| Exchangeable Ca (cmol $kg^{-1}$) | 10.10 | 0.01 |
| Exchangeable Mg (cmol $kg^{-1}$) | 0.26 | 0.07–0.21 |
| CEC (cmol $kg^{-1}$) | 10.33 | 3.86–8.46 |
| Total organic C (%) | 3.37 | 0.57–2.51 |
| Organic matter (%) | 5.81 | nd |
| Total N (%) | 0.13 | 0.04–0.17 |
| Available P (mg $kg^{-1}$) | 2.81 | nd |
| Ammonium (mg $kg^{-1}$) | 25.69 | nd |
| Nitrate (mg $kg^{-1}$) | 21.02 | nd |
| Bulk density (g $cm^{-3}$) | 1.32 | nd |
| Clay (%) | 30 | 16–19 |
| Sand (%) | 56 | 72–76 |
| Silt (%) | 14 | 8–9 |

**TABLE 2**
**Selected Chemical Characteristics of Humin of Composted SW**

| Variables | Value |
|---|---|
| pH | 7.16 |
| Total C (%) | 28.68 |
| Organic matter (%) | 49.45 |
| Total N (%) | 2.16 |
| Total P (mg $kg^{-1}$) | 6076.05 |
| Total K (mg $kg^{-1}$) | 6380.80 |
| CEC (cmol $kg^{-1}$) | 142 |
| Exchangeable Ca (cmol $kg^{-1}$) | 55.23 |
| Exchangeable Mg (cmol $kg^{-1}$) | 24.28 |
| Exchangeable K (cmol $kg^{-1}$) | 65.79 |
| Exchangeable Na (cmol $kg^{-1}$) | 33.36 |

Dry weight of leaf, stem, and root for T5 and T6 was significantly higher than that of T1, T2, T3, and T4. This observation was partly because of humin in T5 and T6 (Table 4).

Observation shows that the relative absorption rates of N and P in maize occurs after 28 days of cultivation, while that of K reaches maximum rate during the early days of planting[27]; hence, the rapid growth of plants of T5 and T6.

**TABLE 3**
**pH Values of Treatments Before and After Fertilizer Formulation**

| Label | Treatment | pH | |
|---|---|---|---|
| | | Without Urea + KCl | With Urea + KCl |
| T3 | (Lquid HA + FA) + NK | 7.46 | 7.43 |
| T4 | Recons. HA + NK | 2.33 | 2.46 |
| T5 | Hydroxide + NK | 10 | 10.66 |
| T6 | (Liquid HA + FA) + NK | 7.47 | 7.46 |

**TABLE 4**
**Dry Weight of Leaves, Stems, and Roots of Maize Plants at 57 DAS**

| Treatment | Dry Weight of Plant (g plant$^{-1}$) | | | |
|---|---|---|---|---|
| | Leaves | Stems | Roots | Total |
| T1 | 2.83a | 1.28a | 1.09a | 5.20a |
| T2 | 1.77a | 0.71a | 0.40a | 2.87a |
| T3 | 2.60a | 1.30a | 0.97a | 4.87a |
| T4 | 2.26a | 1.01a | 1.05a | 4.32a |
| T5 | 15.49b | 22.58b | 6.34b | 44.41b |
| T6 | 12.59b | 17.57b | 5.40b | 35.55b |

Different letters within a column indicate significant difference between means using Tukey's test at $p$ = 0.05.

Except for P, the contents of N and K in leaves and roots were not significant regardless of treatment (Table 5), and this observation could be due to the dilution effect[28]. The P concentration of these parts was significantly higher for T5 and T6 compared to T1. With the exception of P, the contents of N and K of stems for T5 and T6 (treatments with better dry matter production) were not significantly different from T1. This was possible because at critical concentration, growth does not increase to balance the additional nutrient uptake[29].

Treatments with humin (T5 and T6) consistently had significant effect on N, P, and K uptake in leaves, stems, and roots (Table 6) compared to other treatments. A similar observation was made for N, P (except for root), and K use efficiency in leaves, stems, and roots (Table 7). The addition of humin (T5 and T6) possibly acted as a bulky material that enhanced the ability of the soil to resist applied load[30] and increased the porosity of the soil; hence, causing improvement of air and water movement[31]. Compost was found to have a positive effect on crops only when applied with additional N-fertilizer[32], and this was consistent with the findings that T5 and T6 caused better plant growth with half the usual amount of fertilizer (e.g., T2, two times fertilization). Additionally, only 1% of HA was extracted from the composted SW due to mild hydroxide (0.002 M) used for the extraction. Hence, it was considered that humin was still high in HA and FA.

Furthermore, although the fertilization of plants under T5 and T6 was once, total N, P, and K from humin plus inorganic fertilizer provided additional nutrients for the plants. The addition of inorganic fertilizer into compost is a combination of quick and slow release sources, which supplies N throughout the crop growth period. Besides, observation shows that, N, K, and Mg concentrations were high in the soil due to mineralization of humin in the soil[33].

**TABLE 5**
**Effect of Different Treatments on Contents of N, P, and K in Leaves, Stems, and Roots of Maize Plants at 57 DAS**

| Treatment | N | P | K |
|---|---|---|---|
| | | (%) | |
| Leaves | | | |
| T1 | 2.695a | 0.076b | 0.916a |
| T2 | 2.984a | 0.065b | 1.689a |
| T3 | 3.034a | 0.094b | 1.890a |
| T4 | 3.116a | 0.083b | 1.063a |
| T5 | 3.060a | 0.192a | 0.709a |
| T6 | 3.105a | 0.157a | 0.775a |
| Stems | | | |
| T1 | 2.797bc | 0.071c | 2.553c |
| T2 | 4.007a | 0.083bc | 3.682b |
| T3 | 3.960a | 0.125a | 4.626a |
| T4 | 3.534ab | 0.101abc | 4.413ab |
| T5 | 1.956c | 0.138a | 1.742c |
| T6 | 2.399c | 0.124ab | 1.856c |
| Roots | | | |
| T1 | 1.845a | 0.059c | 1.815a |
| T2 | 1.991a | 0.069bc | 2.367a |
| T3 | 2.182a | 0.089abc | 2.458a |
| T4 | 2.285a | 0.075abc | 2.394a |
| T5 | 1.837a | 0.106a | 2.195a |
| T6 | 1.912a | 0.103ab | 2.715a |

Different letters within a column indicate significant difference between means using Tukey's test at $p = 0.05$.

Liquid HA and FA with the addition of humin (T6) in the soil had a remarkable effect on total dry weight (Table 4) and efficiency (Table 7) compared to T3 because FAs have high affinity for mineral chelating and plant growth. They can readily enter plant roots, stems, and leaves, and because of their high exchange capacity as they enter into plant parts, they could carry trace minerals into plant tissues[34]. FAs in acidic condition have the ability to retain $NH_4^+$ ions from urea during hydrolysis[14]. FAs present in low concentration in humic substances have higher acidity than HAs, and thus they are likely to affect stronger soil CEC than HAs[35].

The addition of urea in liquid HA was expected to increase the pH of HA solution, but the result was not as expected. This was because HAs tend to be more aromatic and more prone to precipitation under acidic conditions common in many soils, making them less mobile[36].

Common fertilization by surface application of T2 without any additives (acidic and high CEC materials) can cause nutrients to be easily lost, especially N and K, through volatilization and leaching, but based on the soil characteristics, the soil treated with T2 had quite a high content of N and ammonium compared with other treatments (Table 8). The poor nutrient use efficiency observed for T2 was partly because of poor root development (Table 4). Besides, high Ca in the soil (Table 1) may have reacted with phosphate from the fertilizer to form Ca phosphate, an insoluble compound of phosphate that is generally not available to plants, especially roots. Phosphorus is very important for root development. Stunted roots may affect the whole growth system of a plant negatively. This might be one of the reasons for the poor plant growth, nutrient uptake, and use efficiency observed for plants of T2, T3, and T4.

**TABLE 6**
**Effect of Different Treatments on Uptake of N, P, and K in Leaves, Stems, and Roots of Maize Plants at 57 DAS**

| Treatment | N | P | K |
|---|---|---|---|
| | (mg plant$^{-1}$) | | |
| Leaves | | | |
| T1 | 77.159b | 2.232b | 24.633c |
| T2 | 52.783b | 0.865b | 30.199c |
| T3 | 78.727b | 2.411b | 47.042bc |
| T4 | 69.647b | 1.926b | 24.325c |
| T5 | 473.186a | 29.794a | 109.076a |
| T6 | 397.392a | 20.160a | 95.613ab |
| Stems | | | |
| T1 | 37.010b | 0.918b | 32.471b |
| T2 | 28.712b | 0.555b | 26.312b |
| T3 | 50.460b | 1.583b | 59.175b |
| T4 | 44.458b | 1.020b | 44.212b |
| T5 | 432.334a | 31.376a | 377.954a |
| T6 | 418.864a | 21.815a | 322.223a |
| Roots | | | |
| T1 | 20.786bc | 0.608b | 19.891bc |
| T2 | 8.012c | 0.272b | 9.474c |
| T3 | 20.338bc | 0.881b | 23.522bc |
| T4 | 23.920bc | 0.781b | 26.430bc |
| T5 | 121.404a | 6.838a | 136.161a |
| T6 | 105.894a | 5.653a | 152.599a |

Different letters within a column indicate significant difference between means using Tukey's test at $p = 0.05$.

Among the selected chemical properties analyzed at 57 DAS (Table 8), soil available P was significantly affected by T5 and T6 compared to other treatments. The better growth and development of the maize plants subjected to these two treatments was consistent with this observation. Phosphorus is noted for facilitating good plant root systems, which in turn leads to overall plant growth and development.

## CONCLUSION

Mixing soil with humin produced from composted SW before application of fertilizers (T5 and T6) significantly increased maize dry matter production, N, P, and K uptake. This practice does not only improve N, P, and K use efficiency, but it also helps to reduce the use of N-, P-, and K-based fertilizers by 50%.

## ACKNOWLEDGMENTS

This study was supported by a Fund from the Ministry of Agriculture and Agro-based Industry Malaysia entitled "Utilization of Sago Waste in Animal Production". The authors acknowledge the assistance of the staff of the Universiti Putra Malaysia, Bintulu Sarawak Campus.

**TABLE 7**
**Effect of Different Treatments on N, P, and K Use Efficiency in Leaves, Stems, and Roots of Maize Plant at 57 DAS**

| Treatment | Leaves | Stems | Roots | Total |
|---|---|---|---|---|
| | | % N | | |
| T1 | Nd | Nd | Nd | Nd |
| T2 | 1.574b | 0.682b | 0.708b | 2.957 |
| T3 | 0.888b | 0.675b | 0.500b | 2.064 |
| T4 | 0.357b | 0.491b | 0.466b | 1.314 |
| T5 | 17.751a | 17.720a | 4.510a | 39.981 |
| T6 | 14.354a | 17.116a | 3.815a | 35.283 |
| | | % P | | |
| T1 | Nd | Nd | Nd | Nd |
| T2 | 0.061b | 0.021b | 0.018a | 0.099 |
| T3 | 0.054b | 0.030b | 0.009a | 0.093 |
| T4 | 0.034b | 0.005b | 0.001a | 0.039 |
| T5 | 1.235a | 1.365a | 0.007a | 2.607 |
| T6 | 0.804a | 0.937a | 0.006a | 1.746 |
| | | % K | | |
| T1 | Nd | Nd | Nd | Nd |
| T2 | 1.759bc | 0.931b | 0.904b | 3.594 |
| T3 | 1.624bc | 1.780b | 0.441b | 3.846 |
| T4 | 0.474c | 0.783b | 0.908b | 2.163 |
| T5 | 5.630a | 23.032a | 7.751a | 36.414 |
| T6 | 3.871ab | 19.317a | 8.777a | 31.965 |

Different letters within a column indicate significant difference between means using Tukey's test at $p$ = 0.05. Nd, not determined.

**TABLE 8**
**Soil Selected Chemical Characteristics at 57 DAS**

| | Initial | T1 | T2 | T3 | T4 | T5 | T6 |
|---|---|---|---|---|---|---|---|
| pHKCl | 5.87abc | 5.84abc | 5.74bc | 5.64bc | 5.53c | 6.33a | 6.17ab |
| pHw | 6.45ab | 6.10bcd | 5.99cd | 5.93cd | 5.76d | 6.56a | 6.27abc |
| Exchangeable K (cmol $kg^{-1}$) | 0.132c | 0.129c | 1.458a | 1.118ab | 1.048abc | 0.435cb | 0.499cb |
| Exchangeable Na (cmol $kg^{-1}$) | 0.056d | 0.096cd | 0.174bcd | 0.272bc | 0.160bcd | 0.553a | 0.329b |
| Exchangeable Ca (cmol $kg^{-1}$) | 10.104a | 7.376a | 7.142a | 10.326a | 8.970a | 11.907a | 10.337a |
| Exchangeable Mg (cmol $kg^{-1}$) | 0.264d | 0.463bcd | 0.396cd | 0.642abc | 0.547bcd | 0.888a | 0.723ab |
| CEC (cmol $kg^{-1}$) | 10.330b | 22.222a | 20.333a | 22.000a | 31.056a | 29.778a | 34.833a |
| Total C (%) | 3.368ab | 3.112b | 3.176b | 3.436ab | 3.131b | 3.694a | 3.371ab |
| Organic matter (%) | 5.806ab | 5.366b | 5.476b | 5.925ab | 5.398b | 6.368a | 5.812ab |
| Total N (%) | 0.126a | 0.171a | 0.188a | 0.133a | 0.083a | 0.072a | 0.059a |
| Available P (mg $kg^{-1}$) | 2.807c | 2.113c | 2.239c | 2.688c | 2.693c | 13.126a | 6.854b |
| Ammonium (mg $kg^{-1}$) | 25.685c | 48.257b | 96.124a | 44.365bc | 56.040b | 45.143bc | 49.813b |
| Nitrate (mg $kg^{-1}$) | 21.015b | 36.582a | 45.922a | 41.641a | 45.143a | 36.582a | 43.587a |

Different letters within a column indicate significant difference between means using Tukey's test at $p$ = 0.05.
